# 

**Published:** 1872-03

**Authors:** 


☞ Should any of our subscribers or exchanges fail to get THE COMPANION regularly, they will confer a favor by notifying us of the fact. Our Mailing Clerk will faithfully forward THE COMPANION. Subscribers will please notify Post masters that it will reach their offices, if not lost on the way, between the 15th and 20th of every month. Should it fail to come, after a reasonable time, notify the Editors and it will be sent.

☞ We respectfully solicit Original Articles, Reports of Societies, Clinics Home and Foreign Correspondence, News, etc., etc., of interest to medical readers.

☞ To insure publication, articles should be practical, as brief as possible, and carefully written, on one side of the paper. Articles should be received by the 15th of one month to insure publication in the next. ☞ Friends, send in your articles. ☜

☞ All subscribers and others will please write their names, post-offices, county and State *plainly*. *Remember*, *this is now required by the Postmaster General.*

### PLEASE REMEMBER!

The Publisher desires to remind those of our subscribers who have not yet paid for THE COMPANION of last year, of the fact, and hereby requests them to please remember this appeal, and to *respond at once.* Only a few are in arrears, but we trust that those *few* will not deny themselves the pleasure of evincing their appreciation of the value of THE COMPANION, by remitting at once the small price of subscription. All who have failed to pay for last year will, to direct their attention to the fact, find a *red* cross on this number. Friends, look out for the *red* cross, and remember "just this and no more"—two dollars!

Those who have paid for the year 1872, will have receipts forwarded them just so soon as our clerk can do so. Our subscription list has increased to such an extent that we have been compelled to have a new book sufficiently large to have " room enough for all."

Now is the time to subscribe. In a few weeks not a copy of the January number will be in the Publisher's hands, notwithstanding a heavy edition was issued in view of this large increase of subscribers.

We beg those who have recently subscribed to forward the subscription price promptly. We assure them their copies cost them less than do ours. If our friends will remember the publisher and his little bill, we engage, on our part to give them by far the best journal, for the price, ever issued in this country; while the Publisher will make it, typographically, the equal of any. *The assets of the Companion are larger than those of any other journal of its age on the Continent—if not of many considered* GRAY *in the cause of medical journalism.—Please stick a pin there, and send on your* TWO DOLLARS "*to oil it as it goes.*"

---

### AMERICAN MEDICAL ASSOCIATION.

We have received from the Permanent Secretary, Wm. B. Atkinson, M. D., a notice of the next meeting of this body. We will, in our next, publish the notice. In the meantime, let the friends of the Association everywhere prepare to attend. We learn that our brethren of the city of brotherly love are preparing to extend a hearty welcome to all.

### BOOKS AND PAMPHLETS.

Braithwaite's Retrospect of Practical Medicine and Surgery, Part 64, Jan., 1872, has been received, replete with its usual amount of valuable medical matter. W. A. Townsend, New York. Price, $2.50 in advance; $1.50 half-yearly parts.

The Half-Yearly Abstract of the Medical Sciences; Being a Digest of British and Continental Medicine, etc., etc., edited by Wm. D. Stone, M. D., F. R. C. S., Vol. 54, Jan., 1872, also comes to us with its varied contents of instructive foreign and home medical abstracts. Henry C. Lea, Philadelphia. Price, $2.50; single volume $1.50.

Half-Yearly Compendium of Medical Science, Part 9, Jan., 1872, also lies upon our table—a very valuable retrospect of medical science, especially of our home medical literature. W. S. Butler, M. D., Philadelphia. Price $3.00

The three half-yearly journals above, we regard as indispensible to the scientific, progressive physician; and *all* of them should be in the hands of every *thorough* physician in the land.

The Detection of Criminal Abortion, and a Study of Fœticidal Drugs—By Ely Van De Warker, M. D., Syracuse, N. Y., Corresponding Member of the Gynæcological Society of Boston.

We have before noticed the first pamphlet of Dr. Van De Warker upon Criminal Abortion. The second pamphlet of the series is before us. It will be found highly instructive, and places some facts, in regard to fœticidal drugs before the profession of the greatest value. Dr. Van De Warker is doing a valuable service in behalf of the profession, and one, too, we hope will result in good to the public. James Campbell, Publisher, 18 Tremont street, Boston. Price 50 cents.

Thyrotomy, For the Removal of Laryngeal Growths, Modified. By Ephraim Cutter, M. D., Woburn, Mass. James Campbell, Publisher, Boston.

This monograph will be found both interesting and instructive. Dr. Cutter has modified and improved, we think, the plan of removing growths from the larynx. The old plan was, first to perform tracheotomy, insert the tracheotomy tube, and some days or weeks later, after the larynx had become accustomed to the tube, to perform thyrotomy and remove the growth. Dr. Cutter has abandoned this operation entirely, as well as the use of the tube, and at once performs thyrotomy and removes the growth. Cases are given in which the practicability and success of his plan are shown.

On Vascular Nævi, and Their Treatment by the Actual Cautery. By B. F. Dawson, M. D., New York, Attending Physician to the New York State Woman's Hospital—Out-Door Department," etc., etc.

This is an excellent monograph, from one of the ablest medical writes of the country. Dr. Dawson's monograph will be found a very valuable contribution on the treatment of vascular nævi.

Synopsis and Analysis of One Hundred Cases of Lithotomy, Lithotrity, etc. By Paul F. Eve, M. D., Prof. Practical Surgery, University of Nashville, Tenn.

We thank Prof. Eve for his interesting pamphlet. It is only necessary to give its title, and his name as the author, to insure for the pamphlet the respectful attention of the profession—a respect he so well deserves, and which the profession so delights to award him. May he long live to grace the science of surgery, and to share his knowledge with his brethren.

Annual Report of the Surgeon-General United States Army, 1871.

This Report, which, to be fully understood, and to do justice to Surgeon-General J. K. Barnes, must be read entire. Abounding in statistical facts, no brief notice can do the Report justice, or show the thoroughness and efficiency of the Medical Department of the army under the excellent management of the present Surgeon-General.

Proceedings of the Second Annual Session of the State Medical Association of Arkansas. Held at Litt e Rock, Nov. 5th and 6th, 1871.

We are gratified to notice the high medical status of our brethren of Arkansas, as evinced in the proceedings of the late session of their Medical Association. We took occasion, in our last number to call attention to the suggestions of the President in a matter which, if carried out, will be of service both to the profession and people.

The communications read reflect credit upon the Association no less than upon the authors by whom they were contributed. Dr. R. G. Jennings read a communication giving "a survey of the city of Little Rock." Dr. W. H. Hawkins, one on "Knee-Joint Amputations—two cases reported." Dr. E. R. Du Val, one on a "Case of Malarial Hœmaturia." Dr. Geo. W. Lawrence, on "Climatology: Remarks on the Climatology of the United States." Dr. J. M. Holcombe also read a communication on Hemorrhagic Malarial Fever.

The following officers were elected: President—Dr. J. M. Holcombe; Vice-Presidents—Drs. O. A. Hobson, J. F. Davies, and W. W. Bailey; Recording Secretaries—Drs. E. V. Deuell and Ed. Cross; Corresponding Secretary—Dr. Claiborne Watkins; Treasurer—Dr. J. B. Bond.

We thank Dr. Ed. Cross for the copy of proceedings sent us.

Transactions of the American Otological Society, Fourth Annual Meeting, Newport, R. I., July 19, 1871.

This society is accomplishing a good work. The communications are valuable, and fully up to the times in scientific merit.

The following Reports have been received:

Fourth Annual Report of the New York Orthopædic Dispensary.

Annual Report of the Board of Trustees and Superintendents of the Mississippi State Lunatic Asylum for the year 1871.

Inaugural Address—including a Paper on Infant Asylums—by A. Jacobi, M. D.

The Question of Quarentine: The Natural Prevention of Zymotic Diseases. By Alfred L. Carroll, M. D., New York.

### NOTICES.

Anchor Life Insurance Company.—We call the attention of our medical friends of Georgia to the advertisement of the above named company. As the profession, everywhere, are interested specially in life insurance, we will give our views upon the subject in our next.

Messrs. Berry, Venable & Collier, of Atlanta, have received from Messrs. Tilden & Co. a large supply of their pure extracts and preparations, such as Bromide Sodium, Bromide Calcium, Elixir Pyrophosphate of Iron, Elixir Bromide of Sodium, etc.

Messrs. Redwine & Fox have just received a large supply of pure medicines specially for prescription business, to which they call the attention of their medical friends.

Messrs. W. W. Tucker & Co. make a specialty of compounding prescriptions. Their large supply of pure drugs, and long experience in the prescription business, enable them to render satisfaction to physicians.

